# Expectation and perspectives of patients and physicians on new treatment for early Alzheimer Disease: Results of a physician survey and patient focus group interview

**DOI:** 10.1002/alz.086956

**Published:** 2025-01-09

**Authors:** Joanne Bell, H. Ricky Kurzman, Stephane Borentain, Russ Paulsen, Andrew Holzapfel, Harald Hampel

**Affiliations:** ^1^ Eisai Inc., Nutley, NJ USA; ^2^ Eisai, Nutley, NJ USA; ^3^ EISAI Inc, NUTLEY, NJ USA; ^4^ UsAgainstAlzheimer’s, Washington, DC USA; ^5^ Davos Alzheimer’s Collaborative, Geneva Switzerland

## Abstract

**Background:**

With the recent approval of disease modifying therapies (DMT) for early Alzheimer’s disease, there is a need for prescribing physicians to accurately communicate expectations of treatment effects to patients and their care partners. To better understand potential challenges and solutions to enhance this communication, physicians were surveyed, after which patients and care partners participated in focus groups.

**Method:**

Step one consisted of an online survey of 100 US‐based neurologists, geriatric medicine specialists and Alzheimer’s disease specialists to ascertain physician perspectives on meaningful benefits associated with anti‐amyloid monoclonal antibody treatment (DMT) over currently available symptomatic treatments. Informed by the physician survey findings, six focus groups collected qualitative narratives from patients and their care partner(s). The objectives were to explore patient and care partner treatment goals, their experience with symptomatic treatment and their expectations for new therapies.

**Results:**

Physicians reported that the main concerns for patients and their care partner are memory loss (84%) behavioral and mood changes (51%) and ability to complete daily activities (51%). Most physicians expected a DMT to be effective in slowing the disease progression, and 66% expected to see cumulative benefit over time with continued treatment. Physicians reported that being able to perform activities of daily living (84%), maintaining quality of life (79%) and remaining independent (77%) are the most meaningful treatment outcomes for patients. Physician expectations were largely similar between symptomatic treatment and DMT. During the focus groups, patients and care partners noted the impact on financial status, social interaction, and loss of independence to be the three major areas of concern associated with their diagnosis. Focus group participants noted that maintaining their current state longer is a meaningful treatment outcome and prioritized three domains: effective communication, thinking clearly and less fear while performing daily activities.

**Conclusion:**

This research suggests the need for a better physician/patients‐care partner communication on appropriate treatment expectations and for the development of tools to facilitate this communication. Findings from this research need to be confirmed by a large survey of a nationally representative sample of US patients.

